# Potential miRNA biomarkers and therapeutic targets for early atherosclerotic lesions

**DOI:** 10.1038/s41598-023-29074-1

**Published:** 2023-03-01

**Authors:** Genesio M. Karere, Jeremy P. Glenn, Ge Li, Ayati Konar, John L. VandeBerg, Laura A. Cox

**Affiliations:** 1 Department of Internal Medicine, Section on Molecular Medicine, Center for Precision Medicine, Wake Forest School of Medicine, Winston-Salem, NC 27157 USA; 2 Southwest National Primate Research Center, Texas Biomedical Research Institute, San Antonio, TX 78227 USA; 3 Department of Human Genetics, South Texas Diabetes and Obesity Institute, The University of Texas Rio Grande Valley, Brownville, Harlingen, Edinburg, TX 78520 USA

**Keywords:** Genetics, Non-coding RNAs, miRNAs

## Abstract

Identification of potential therapeutic targets and biomarkers indicative of burden of early atherosclerosis that occur prior to advancement to life-threatening unstable plaques is the key to eradication of CAD prevalence and incidences. We challenged 16 baboons with a high cholesterol, high fat diet for 2 years and evaluated early-stage atherosclerotic lesions (fatty streaks, FS, and fibrous plaques, FP) in formalin-fixed common iliac arteries (CIA). We used small RNA sequencing to identify expressed miRNAs in CIA and in baseline blood samples of the same animals. We found 412 expressed miRNAs in CIA and 356 in blood samples. Eight miRNAs (miR-7975, -486-5p, -451a, -191-5p, -148a-3p, -17-5p, -378c, and -144-3p) were differentially expressed between paired fatty streak lesion and no-lesion sites of the tissue, and 27 miRNAs (e.g., miR-92a-3p, -5001, -342-3p, miR-28-3p, -21-5p, -221-3p, 146a-5p, and -16-5p) in fibrous plaques. The expression of 14 blood miRNAs significantly correlated with extent of lesions and the number of plaques. We identified coordinately regulated miRNA-gene networks in which miR-17-5p and miR-146a-5p are central hubs and miR-5001 and miR-7975 are potentially novel miRNAs associated with early atherosclerosis. In summary, we have identified miRNAs expressed in lesions and in blood that correlate with lesion burden and are potential therapeutic targets and biomarkers. These findings are a first step in elucidating miRNA regulated molecular mechanisms that underlie early atherosclerosis in a baboon model, enabling translation of our findings to humans.

## Introduction

Atherosclerotic cardiovascular disease (CVD) is the leading cause of morbidity and mortality globally, especially in developed countries^1^. Atherogenesis can be defined as a result of gene and protein expression changes leading to altered cellular processes and remodeling of local blood vessels. Atherogenesis is a complex progressive process involving many cellular processes in vascular cells, including endothelial cells, smooth muscle cells, and platelets, and in macrophages and innate immune cells^2–5^. Many central mechanisms are associated with atherogenesis, but the most compelling, include the modification of LDL via oxidation, desialylation of lipoproteins; and particle size alteration; activation and injury of vascular endothelial cells and infiltration of modified LDL into the intimal layer; formation of foam cells; production of cytokines and inflammation cascade; mitochondria dysfunction; dedifferentiation of smooth muscle cells; and autophagy impairment leading to accumulation of cell debris and plaque formation^5–9^.

Many human studies are focused on late-stage anatomical changes that are evident long after these initial molecular changes partially because of availability of arterial tissues after surgery^10,11^. The key to reducing CVD mortality and morbidity is early detection and treatment of atherosclerosis prior to irreversible tissue changes with advancement to life-threatening late-stage plaques^12–14^. To achieve this goal, there is a need to better understand the molecular mechanisms underlying the changes that lead to initiation and progression of atherosclerosis for prevention and treatment.

We posit that microRNAs (miRNAs) play a regulatory role in gene and protein expression changes observed during atherogenesis, and that miRNA related molecular mechanisms underlie initiation and progression of atherosclerosis. miRNAs are small non-coding RNAs that regulate gene expression post-transcriptionally by binding mRNA untranslated regions, resulting in degradation of the mRNA or inhibition of translation^15^. The first step in deciphering these miRNA related mechanisms linked to CVD is to compare miRNA expression in early atherosclerotic lesions with healthy sites of arteries and identify gene targets of these miRNAs as well as miRNA-gene networks involved.

The baboon model of atherosclerosis presents opportunities to investigate early molecular changes leading to development of atherosclerosis. It is not tenable to obtain arterial tissues from apparently healthy humans to decipher initial molecular changes accompanying early atherosclerosis. Baboons develop atherosclerosis naturally like humans^8^; unlike murine models, baboons do not require genetic manipulation to render them susceptible to atherosclerosis^16^. Non-human primates share similar genetic and physiological characteristics, including those involved in lipid metabolism and immunity with humans^17^. Therefore, understanding miRNA molecular mechanisms underlying atherosclerosis initiation in non-human primates is translatable to humans. The goal of our study is to provide novel insights to miRNA-related mechanisms in early atherosclerotic lesions in baboons. To our knowledge this is the first study to do so in non-human primates, and it provides a foundation to understanding atherosclerosis initiation as a basis for early detection and intervention in humans.

We previously challenged baboons with a high cholesterol, high fat (HCHF) diet for 2 years to stimulate early atherosclerosis (n = 112). We observed that HCHF diet stimulated increase of atherogenic markers and development of atherosclerotic lesions in common iliac arteries (CIA), a surrogate of systemic atherosclerosis^18,19^, with extensive variation in lesions among study animals^19^. Further, we evaluated the characteristics of these lesions, corresponding to fatty streaks and fibrous plaques^20^. In the current study, we quantified miRNAs expressed in the lesions and no-lesion sites of the CIA, and in blood samples. Comparing lesion and no-lesion sites of the same tissue of individual animals enables complete control of genetic and environmental factors that might influence miRNA expression. Using Ingenuity Pathway Analysis (IPA), we identified predicted miRNA targets and miRNA-gene networks that are central to deciphering molecular mechanisms underlying initiation and progression of atherosclerosis in primates. Finally, we discuss miRNAs expressed in atherosclerotic vasculature in humans and in baboons.

## Materials and methods

### Humane care guidelines

All research procedures involving animals for this study were conducted in facilities certified by the Association for Assessment and Accreditation of Laboratory Animal Care (AAALAC) at the Southwest National Primate Research Center (SNPRC) in San Antonio, Texas by board certified veterinarians and veterinary staff. SNPRC veterinarian euthanized animals using ketamine and pentobarbital followed by exsanguination in accordance with American Veterinary Medical Association (AVMA) and Animal Research: Reporting of In Vivo Experiments (ARRIVE) guidelines and were approved by the Institutional Animal Care and Use Committee (IACUC) at the Texas Biomedical Research Institute, the host institution for the SNPRC.

### Diet challenge and tissue collection

In previous work, 112 adult baboons (47 females and 65 males) were challenged with HCHF diet for 2 years and tissues were collected at the end of the study. Blood samples were collected at the beginning (baseline) and end of the study. Mean age of the females was 12.6 years (range = 8.7–17.0 years) and that of the males was 11.4 years (range = 8.1–14.1 years). Study details and diet composition have been described^19^.

CIA were collected and preserved in 10% formalin. The CIA were defatted and dissected longitudinally and stained as described^19,21^. The lesion type and the extent (proportion of tissue surface area covered by lesions) of atherogenic lesions was evaluated as described^19^. The lesion types were validated by immunohistochemistry^20^.

For the current study, a subset of CIA was selected from the 112 samples that represented the two categories of lesion type: fatty streaks (FS) and fibrous plaques (FP). The characteristics of the samples included 3 males and 5 females for FS, and 4 males and 4 females for FP. Mean age of the females was 12.0 years (range = 11.1–12.5 years) and that of the males was 11.2 years (range = 11.6–10.9 years).

### Total RNA isolation

For the same animals, we isolated total RNA from baseline blood samples as described^22^ and from CIA using RecoverAll kit (Ambion). Briefly, we excised paired lesion and no-lesion sites of CIA from each tissue sample. We homogenized the tissues in lysis buffer using a Mini-Beadbeater-96 (Biospecproducts) for 5 min. The lysates were processed using the kit according to manufacturer’s protocol. RNA was quantified using Qubit Fluorimeter (DeNovix).

### miRNA sequencing and sequence analysis

These steps were performed as described previously^22^. Briefly, for each sample, we used 500 ng of total RNA to generate cDNA libraries using NextFlex Small RNA-Seq Kit v3 (PerkinElmer). After cDNA quality check, two sets of 24 samples each were pooled and quantified. We multiplexed cDNA into two pools, each containing 24 samples, and hybridized the pool to an individual lane of the flow cell. We performed small RNA sequencing using Illumina HiSeq 2500 instrument and reagents, including HiSeq Rapid Cluster v2 and HiSeq Rapid SBS Kit v2 (50 cycles) for cluster generation. We used mirDeep2 pipeline to analyze fastq-formatted sequence reads with a Phred quality score of 30 or greater and an inferred base call accuracy of 99.9% to identify miRNA sequences and assess expression levels (read counts). miRNA read counts were normalized holistically using reads per million mapped to miRNA.

### miRNA target identification, miRNA-gene networks, and pathway analysis

These steps were performed as described previously^22^. Briefly, differentially expressed miRNAs were imported to IPA and Core analysis performed to identify miRNA target genes related to cardiovascular system signaling and to generate networks. Putative miRNA target genes were identified using TargetScan embedded in IPA. Subsequently, we used miRNA targets predicted with high confidence and experimentally validated targets, to identify miRNA-gene networks.

### Statistical analysis

We used the One-Way analysis of variance tool embedded in Partek Genomics Suite (Partek Inc.) to compare differences in variable distribution between lesion and no-lesion regions of CIA per lesion type and between lesion type. We used Prism 8 (GraphPad) to perform Pearson’s correlation analysis and identify circulating miRNAs expressed at baseline that associated with the extent of atherosclerosis measured after 2-year diet challenge. Statistical significance was tested with *p* < 0.05, and all tests were two-tailed. Multiple testing correction was performed using FDR q-value < 0.05.

## Results

### Number of expressed miRNAs

We analyzed miRNAs expressed in 8 pairs (lesion and no-lesion) of CIA with FS and 8 pairs with FP, collected after 2 years of diet challenge. In addition, we analyzed miRNAs expressed in blood samples from the same animals, collected at baseline prior to the 2 years diet challenge. We observed that 412 miRNAs were expressed in CIA (passing quality filters). Three hundred fifty-six miRNAs were expressed in blood samples (passing quality filters). Two hundred ninety-two miRNAs were expressed in both CIA and blood samples.

### Differentially expressed miRNAs

We identified miRNAs with differing expression between lesion and non-lesion regions of CIA. In FS samples, 9 miRNAs were significantly up-regulated compared to the no-lesion site of the tissue (Table 1). For FP samples, 27 miRNAs (17 up-regulated and 10 down-regulated) were differentially expressed compared to no-lesion site of the tissue (Table 2).

**Table 1:** Differentially expressed miRNAs between FS and no lesion regions in baboon CIA after 2-year diet challenge.

ID	*p*-value	q-value	Fold-Change
pha-miR-7975	1.99E−10	7.26E−08	8.6
pha-miR-486-5p	8.27E−06	1.51E−03	4.6
pha-miR-144-3p	8.68E−04	4.52E−02	3.3
pha-miR-191-5p	3.28E−05	3.98E−03	3.2
pha-miR-451a	8.65E−04	4.52E−02	3.1
pha-miR-106b-5p	6.41E−04	4.52E−02	3.0
pha-miR-148a-3p	8.64E−04	4.52E−02	2.9
pha-miR-93-5p	1.18E−03	4.79E−02	2.5
pha-miR-378c	1.08E−03	4.79E−02	2.1

Fold-change is defined as the ratio of the average expression level of a miRNA in FP lesion and in no-lesion control and log2 transformed. Negative fold-change values were calculated as –(1/Fold-change). *p* < 0.05, q < 0.05.

**Table 2:** Differentially expressed miRNAs between FP and no lesion regions in baboon CIA after 2-year diet challenge.

ID	*p*-value	q-value	Fold-Change
pha-miR-451a	2.12E−05	1.18E−03	7.8
pha-miR-146a-5p	5.55E−05	1.92E−03	6.8
pha-miR-7975	1.15E−09	3.83E−07	6.7
pha-miR-191-5p	8.86E−07	1.46E−04	6.1
pha-miR-486-5p	1.32E−06	1.46E−04	5.9
pha-miR-144-3p	1.11E−05	7.42E−04	5.8
pha-miR-106b-5p	1.54E−04	4.32E−03	4.5
pha-miR-17-5p	5.74E−05	1.92E−03	4.2
pha-miR-93-5p	1.55E−04	4.32E−03	4.2
pha-miR-342-3p	1.47E−03	2.26E−02	3.9
pha-miR-25-3p	1.17E−03	2.05E−02	3.7
pha-miR-222-3p	4.19E−05	1.75E−03	2.8
pha-miR-221-3p	2.25E−03	2.78E−02	2.6
pha-miR-21-5p	8.19E−04	1.61E−02	2.4
pha-miR-92a-3p	1.66E−03	2.31E−02	2.4
pha-let-7g-5p	2.18E−03	2.78E−02	1.5
pha-let-7f-5p	9.71E−04	1.80E−02	1.3
pha-miR-24-3p	3.15E−04	7.01E−03	− 1.4
pha-miR-145-5p	1.75E−03	2.33E−02	− 1.5
pha-miR-143-3p	2.59E−04	6.16E−03	− 1.5
pha-miR-23b-3p	4.02E−04	8.38E−03	− 1.6
pha-miR-28-3p	1.49E−03	2.26E−02	− 2.0
pha-miR-143-3p	1.04E−05	7.42E−04	− 2.0
pha-miR-5100	3.20E−05	1.52E−03	− 2.1
pha-let-7e-5p	3.10E−03	3.69E−02	− 2.1
pha-miR-195-5p	1.64E−03	2.31E−02	− 2.3
pha-miR-23c	1.49E−03	2.26E−02	− 4.1

Fold-change is defined as the ratio of the average expression level of a miRNA in FP lesion and in no-lesion control and log2 transformed. Negative fold-change values were calculated as –(1/Fold-change). *p* < 0.05, q < 0.05.

### Lesion-specific expressed miRNAs

**Figure 1:**
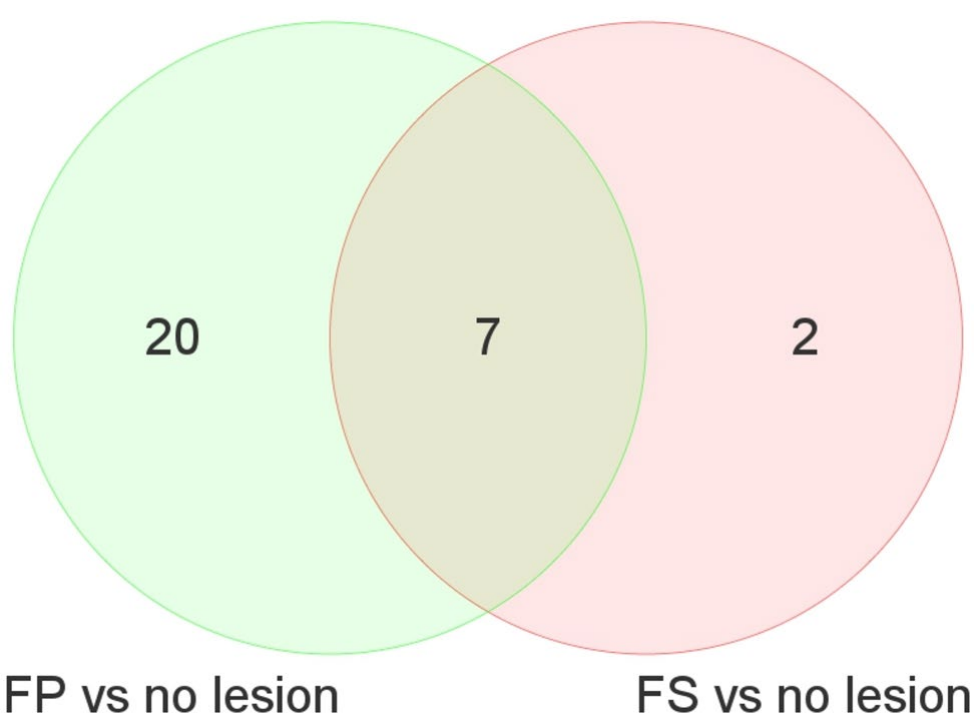
Number of miRNAs differentially expressed between lesion and no lesion sites of baboon CIA.

Two miRNAs (miR-148a-3p and miR-378c) up-regulated were unique to FS lesions. Twenty miRNAs (10 up-regulated; miR-222-3p, miR-221-3p, miR-17-5p, miR-21-5p, miR-7f-5p, miR-25-3p, miR-342-3p, miR-92a-3p, miR-7e-5p, miR-a-5p and 10 down-regulated; miR-143-5p, miR-5100, miR-1461-5p, miR-143-3p, miR-24-3p, miR-23b-3p, miR-23c, miR-28-3p, miR-195-5p, miR-145-5p) were uniquely expressed in FP lesions. Seven miRNAs (miR-7975, miR-486-5p, miR-144-3p, miR-191-5p, miR-106b-5p, miR-93-5p, miR-451a) were upregulated in both FS and FP when compared to the no-lesion site of the tissue (Fig. 1). miRNAs expressed in FS or in FP did not show significant sex differences after adjusting for multiple testing, q < 0.05.

### Circulating miRNAs that correlate with extent of lesions

We performed correlation analysis to identify circulating miRNAs in blood samples collected at baseline that correlated with extent of atherosclerosis. For the study cohort, the average extent of early atherosclerosis was 14% and ranged from 6 to 77%. The average number of plaques was 1, ranging from 1 to 7. We identified 14 miRNAs that significantly correlated with extent of CIA atherosclerotic lesions and the number of plaques (Table 3). Four miRNAs (miR-30d-5p, miR-21-5p, miR-339-5p, and miR-1301-3p) uniquely predicted extent of FS lesions, three miRNAs (miR-30a-5p, miR-30c-5p, and 5699-5p) uniquely predicted extent of FP, and miR-3200-3p predicted number of plaques. Two miRNAs (miR-30a-3p and miR-10527-5p) predicted both the extent and number of FP lesions.

**Table 3:** Baseline expressed circulating miRNAs that significantly correlate with extent of fatty streaks, fibrous plaques, and the number of plaques in baboon CIA after 2-year diet challenge.

Baseline blood miRNAs	Extent of FS	Extent of FP	Number of plaques
pha-miR-30a-5p	0.06 (− 0.36)	0.03 (− 0.40)*	0.06 (− 0.36)
pha-miR-30c-5p	0.19 (− 0.25)	0.04 (− 0.39)*	0.06 (− 0.35)
pha-miR-486-5p	0.03 (0.41)*	0.11 (0.31)	0.03 (0.41)*
pha-miR-5699-5p	0.29 (− 0.21)	0.04 (− 0.38)*	0.09 (− 0.32)
pha-miR-30a-3p	0.14 (− 0.29)	0.01 (− 0.46)*	0.03 (− 0.41)*
pha-miR-3200-3p	0.05 (− 0.29)	0.1 (− 0.46)	0.04 (− 0.41)*
pha-miR-10527-57	0.29 (− 0.21)	0.01 (− 0.48)*	0.02 (− 0.42)*
pha-miR-873-3p	0.02 (0.43)*	0.20 (− 0.48)	0.04 (− 0.42)*
pha-miR-3155a	0.02 (0.42)*	0.14 (0.28)	0.02 (− 0.42)*
pha-miR-3155b	0.02 (0.42)*	0.13 (0.29)	0.02 (0.42)*
pha-miR-30d-5p	0.02 (− 0.43)*	0.42 (− 0.15)	0.38 (− 0.17)
pha-miR-21-5p	0.04 (− 0.26)*	0.28 (− 0.21)	0.33 (− 0.19)
pha-miR-339-5p	0.02 (− 0.42)*	0.09 (− 0.32)	0.14 (− 0.28)
pha-miR-1301-3p	0.04 (0.38)*	0.05 (0.37)	0.16 (0.27)

The numerals in the second to fourth columns depict *p* values and in parathesis are Pearson’s correlation coefficient (r) values. The asterisk indicates a correlation that reached a significant level, *p* < 0.05.

### miRNA targets and miRNA-gene networks

**Figure 2:**
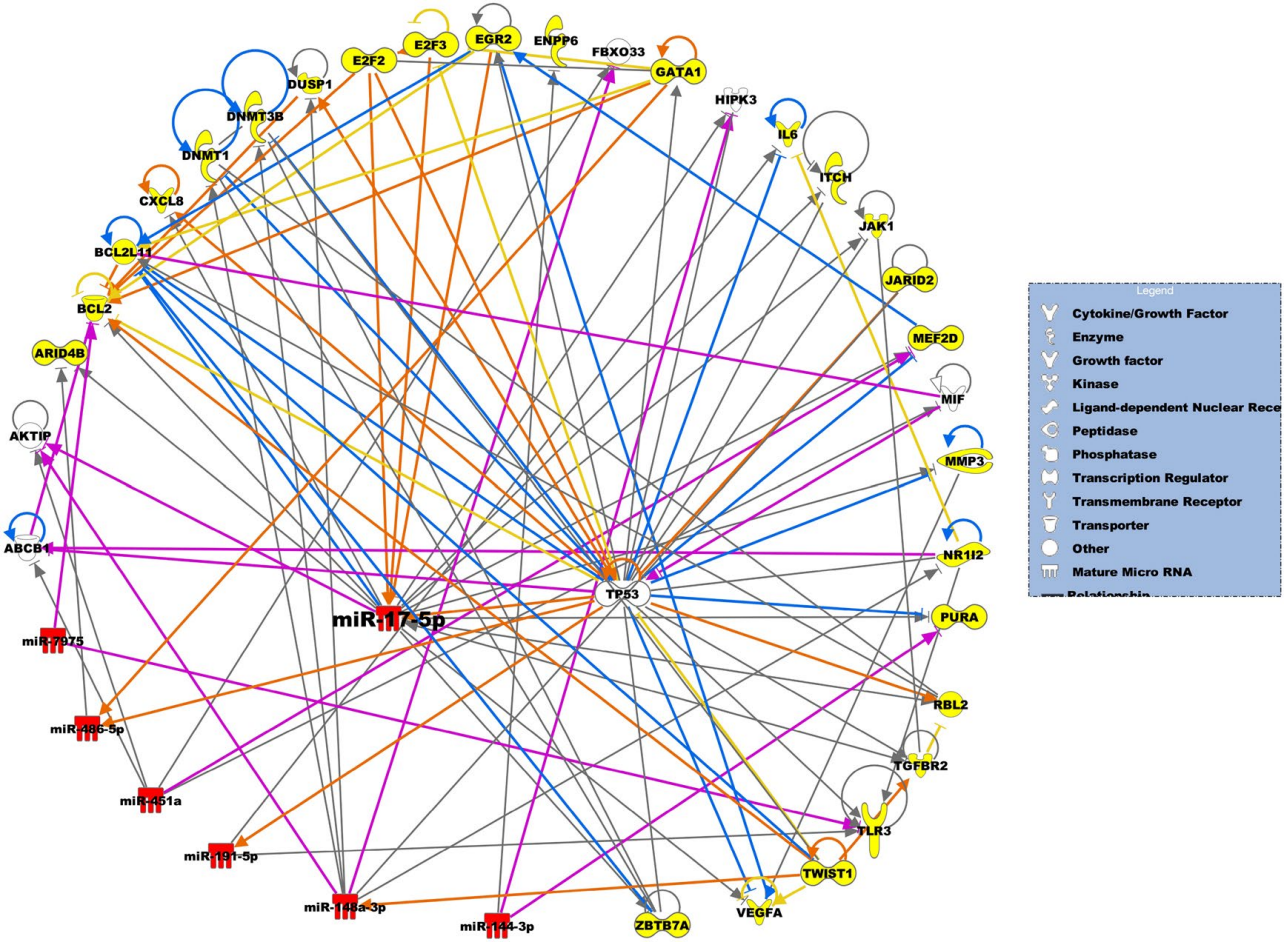
miRNA-gene network that underlie development of FS lesions. miRNAs and predicted miRNA target genes are represented as nodes. Red nodes indicate upregulated miRNAs. Yellow nodes depict miRNA target genes unique to FS lesions. The molecular relationships between nodes is presented as a line (edge); arrows indicate the direction of direct interaction.

**Figure 3:**
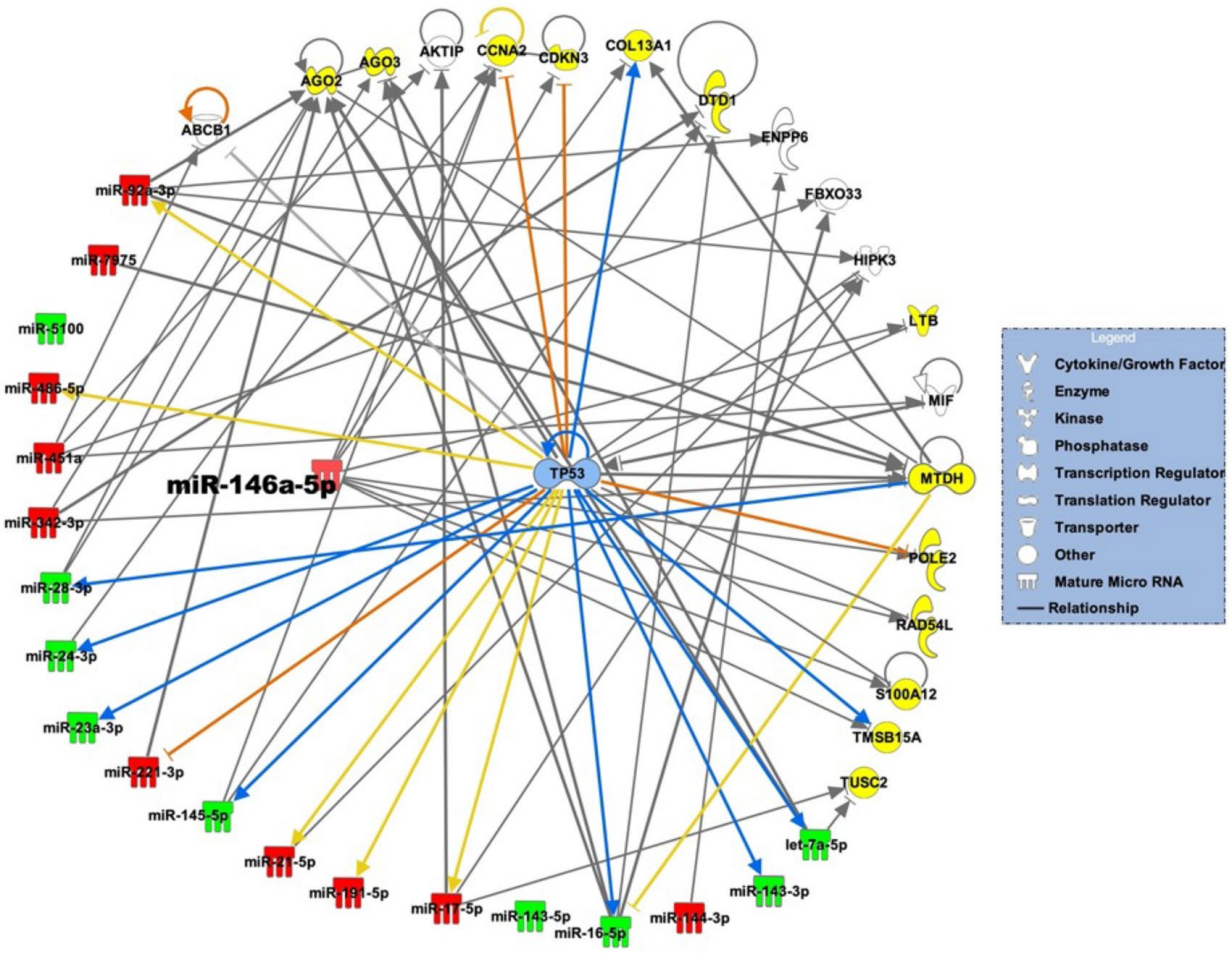
miRNA-gene network that underlie development of FP lesions. miRNAs and predicted miRNA target genes are represented as nodes. Red and green nodes indicate, respectively, upregulated and downregulated miRNAs. Yellow nodes depict miRNA target genes unique to FP lesions. The molecular relationships between nodes is presented as a line (edge); arrows indicate the direction of direct interaction.

We identified genes predicted to be targeted by the differentially expressed miRNAs. Supplemental Table 1 shows the miRNA targets. Figures 2 and 3 show the miRNA-gene networks, respectively, related to FS and FP lesions. For the FS network, 25 putative genes are targeted by differentially expressed miRNAs, including IL6, MMP3, TGFBR2, TLR3, BCL2, CXCL8 and VGFA, and miR-17-5p is the central regulatory molecule. For the FP network, differentially expressed miRNAs target 13 genes, including CCNA2, CDKN3, COL13A1, DTD1, POLE2, and TUSC2, and miR-146a-5p is the central regulatory molecule. Seven genes are targeted by differentially expressed miRNAs in both FS and FP lesions, including TP53, ABCB1, FBXO33, AKTIP, ENPP6, HIPK3, and MIF.

## Discussion

Identification of miRNAs expressed in relevant tissues and gene targets is essential for providing insights into molecular mechanisms underlying initiation and progression of atherosclerosis. In this study we analyzed baboon CIA samples with early-stage atherosclerotic lesions, including FS, which are initial lesions, and FP, which are relatively more advanced than FS^20^. We identified miRNAs differentially expressed between paired lesion and no-lesion sites in the vascular tissue, putative genes targeted by the differentially expressed miRNAs, and miRNA-gene networks specific to the lesion types. In addition, we identified potential circulating miRNA biomarkers predicting burden of atherosclerosis. To our knowledge, this is the first study to identify miRNAs expressed in vascular tissues with early atherosclerotic lesions and their putative targets in a non-human primate model of atherosclerosis. Our findings provide critical insights to identifying potential miRNA related molecular mechanisms that underlie early atherosclerosis in primates.

In our study we identified miRNAs that are differentially expressed between lesion and no-lesion sites of CIA and that which are lesion specific. Differentially expressed miRNAs include 8 miRNAs (miR-7975, miR-486-5p, miR-451a, miR-191-5p, miR-148a-3p, miR-17-5p, miR-378c, and miR-144-3p) in FS lesions, and 27 miRNAs (e.g., miR-92a-3p, -5001, -342-3p, miR-28-3p, -21-5p, -221-3p, and -16-5p) in FP lesions. Two miRNAs (miR-148a-3p and miR-378c) were uniquely expressed in FS lesions and 20 miRNAs (miR-143-5p, miR-5100, miR-222-3p, miR-1461-5p, miR-17-5 pm, miR-106a-5p, miR-143-3p, miR-24-3p, miR-23b-3p, miR-21-5p, miR-7f-5p, miR-25-3p, miR-342-3p, miR-23c, miR-28-3p, miR-195-5p, miR-92a-3p, miR-145-5p, miR-221-3p, miR-7e-5p) were uniquely expressed in FP lesions. Our findings show that some miRNAs are not only lesion-type specific but exhibit differential expression in different stages of the disease indicating relevance in disease initiation and progression.

Previous in vitro studies have showed that some of the differentially expressed, lesion-specific miRNAs identified in this study are involved in atherogenesis. For example, miR-143, miR-221 and miR-146a promote proliferation of vascular smooth cells^23–25^, a hallmark of initiation of atherogenesis. In contrast, miR-221 promotes apoptosis in endothelial cells by targeting PGC-1alpha resulting in mitochondria dysfunction and accumulation of RO species^26^. miR-92a is upregulated by shear stress and expressed in regions that are athero-susceptible^27^. In macrophages, miR-106, miR-378, miR-148a inhibit cholesterol efflux by cooperatively targeting ABCA/G1^28–30^.

We have identified vascular miRNAs expressed in baboons and in humans and potentially novel miRNAs associated with atherosclerosis. For example, we observed that miR-144-3p, miR-146a-5p, miR-21, miR-221/222-3p are up-regulated in FP lesions and miR-195-5p was downregulated. In human studies, miR-21 and miR-146a-5p were up-regulated in carotid and aortic lesions^11^ while miR-144-5p and miR-221-3p were up-regulated and miR-195-5p down-regulated in tissues with aortic aneurysm compared to control^31^. Currently miR-195 is in preclinical trial for treatment of post-myocardial infarction^32^. These human studies collaborate our baboon findings. Moreover, we identified potentially novel miRNAs expressed in CIA early atherosclerotic lesions, including miR-7975and miR-5001. In our study miR-7975 exhibited high fold changes, respectively, 8.6 and 6.7 in FS and in FP lesions. Previous studies have shown that miR-7975 is involved in regulation of lung inflammation and in lung cancer^33,34^. In addition, a previous study indicated that miR-5001 expression was low in colorectal tumors and downregulation of miR-5001 that targets HES6 gene promoted cell proliferation^35^. Recent studies suggest miR-5001 is a potential biomarker for prostate and ovarian cancer^36,37^. Appreciating that the expression of miRNAs is tissue and/or cell specific, the role of miR-7975 and miR-5001 in development of atherosclerosis is not known and should be investigated in future studies.

miRNAs are potential biomarkers indicative of disease and health status and responsiveness to therapeutic interventions^38–43^. Findings from our study indicate that 14 circulating miRNAs are potential biomarkers of disease progression and lesion number, predicting the burden of CIA atherosclerosis. Of the 14 miRNAs, four (miR-21-5p, miR-339-5p, miR-30d-5p, and miR-1301-3p) correlated specifically with extent of FS lesions. Two miRNAs (miR-30a-3p and miR-10527-5p) correlated specifically with both the extent and number of fibrous plaques in CIA. Previous human studies have shown that miR-21-5p is overexpressed in serum of patients with coronary artery disease^44–46^. miR-339-5p and miR-1301-3p regulates smooth cell proliferation^47,48^ but to the best of our knowledge their role as biomarkers of human atherosclerosis is not known. Similarly, the role of miR-30d-5p, miR-30a-3p, and miR-10527-5p as biomarkers of human atherosclerosis is not known. Thus, our study presents potential novel biomarkers of early atherosclerosis.

We identified coordinately regulated miRNA-gene networks associated with early atherosclerotic lesions. The miRNA-gene networks play a critical role in putting large datasets into biological context for dissemination of potential hypothesis and molecular mechanisms related to disease status. For the FS network, among the seven miRNAs that target 25 genes, miR-17-5p is predicted to regulate most of the genes (13), including BCL2, VEGFA, CXCL8, IL6, MMPs, JAK1, ITCH, TLR3, and TGFBR2, which were previously demonstrated to be associated with atherosclerosis. For example, BCL2, encodes an outer mitochondrial membrane protein that is anti-apoptotic. The molecule is downregulated in aortic atherosclerotic lesions in rats^49^. VEGFA is a growth factor that induces proliferation and migration of vascular endothelial cells, and is essential for both physiological and pathological angiogenesis, resulting in plaque neovascularization, increased microvessel permeability, and intraplaque hemorrhage^50,51^. This process is linked to plaque progression through the accumulation of erythrocyte membranes, which are rich in free cholesterol and promote necrotic core enlargement^52^. CXCL8/IL-8 is a member of the CXC chemokine family and is a major mediator of the inflammatory response, a potent angiogenic factor, associated with development of atherosclerosis^53^. Although these genes are associated with atherogenesis, the mechanistic role of miR-17-5p on expression of these genes and involvement in the disease development is not known.

In addition, genes predicted to be targeted by the miR-17-5p, not previously associated with atherosclerosis, include ARID4B, E2F, GATA1, MEF2D, NR1I2, PURA, and RBL2. For example, ARID4B, encodes a protein with sequence similarity to retinoblastoma-binding protein-1, which functions in diverse cellular processes including proliferation, differentiation, apoptosis, oncogenesis, and cell fate in cancer cells. The expression of this gene was associated with air-pollution induced methylation in Multi-Ethic Study of Atherosclerosis (MESA) participants^54^. E2F, is a member of the E2F family of transcription factors. The E2F family plays a crucial role in the control of cell cycle and action of tumor suppressor proteins and is also a target of the transforming proteins of small DNA tumor viruses. This protein is involved in regulation of smooth muscle cell cycle^55^. We observe that these are potentially novel genes associated with development of atherosclerosis.

For the FP network, among the 19 miRNAs that target 13 genes, miR-146a-5p is the central hub predicted to regulate majority of the genes (8), including CCNA2, CDKN3, COL13A1, LTB, POLE2, RAD54L, S100A12, TUSC2, and TMSB15A. These genes have not previously been associated with development of atherosclerosis. The interactions of these genes with miR-146a-5p in relation to atherogenesis are not known. For example, CCNA2 protein encoded by this gene belongs to the highly conserved cyclin family, whose members function as regulators of the cell cycle. This protein binds and activates cyclin-dependent kinase 2 and thus promotes transition through G1/S and G2/M. CDKN3, is a cyclin-dependent kinase inhibitor, and has been shown to interact with, and dephosphorylate CDK2 kinase to regulate cell cycle. The gene was downregulated after exposure to cigarette smoke condensate^56^. Previous work demonstrated that miR-146a-5p interacts with CDKN3 in clear cell renal cell carcinoma^57^. COL13A1, is a collagen protein expressed in plasma membrane of connecting tissue-producing cells. Its function is not known. POLE2 is a DNA polymerase epsilon, which is involved in DNA repair and replication, is composed of a large catalytic subunit and a small accessory subunit. Defects in this gene have been linked to colorectal cancer and to combined immunodeficiency^58^. TUSC2 encodes a tumor suppressor 2 protein that is deleted in some carcinomas, including lung cancer. TUSC2 elicits its anti-tumor effects through regulating G1 cell cycle progression, apoptosis, calcium homeostasis, gene expression, and the activity of various protein tyrosine kinases and Ser/Thr kinases^59^.

Our findings suggest that miR-17-5p and miR-146a-5p, which are up regulated in FS and FP lesions, respectively, may be key molecules involved in molecular mechanisms that underlie initiation and progression of early atherosclerosis. Previous studies have demonstrated that miR-17-5p and miR-146a-5p are associated with atherosclerosis^44,60–62^. The miR-17-5p is activated in macrophages and promotes development of lesions. Silencing of this miRNA reduces lipid accumulation and production of inflammatory cytokines in vitro and in vivo to ameliorate atherosclerosis^62^. In addition, miR-17-5p is a circulatory biomarker of coronary atherosclerosis^60^. In contrast, miR-146a-5p has been shown to alleviate inflammation in endothelial cells and to reduce atherosclerosis in mice^61^. Our finding that miR-146a-5p is upregulated in lesions contradicts results from previous cellular and mouse studies but corroborate results of a study indicating that plasma miR-146a-5p is upregulated in human patients with myocardial infarction compared to control^44^. Our future studies will focus on the validation of the interactions between the miRNAs and miRNA targets identified in this study, and the identification of the molecular mechanisms by which miRNAs regulate the pathway leading to the development of atherosclerosis. These findings suggest new insights to potential roles of miRNAs in initiation and progression of atherosclerosis, and potential targets for early interventions.

## Conclusion

For the first time, we have identified miRNAs that correlate with the types and extent of early atherosclerotic lesions in baboons and coordinated regulated miRNA-gene networks associated with early atherosclerosis. We identified 8 miRNAs that are differentially expressed in FS and 27 in FP lesions and novel miRNA targets associated with early atherosclerosis. We revealed that miR-17-5p and miR-146a-5p are potential gene expression regulatory hubs associated, respectively, with initiation and progression of early atherosclerotic lesions in a baboon model of atherosclerosis. In addition, we identified potentially novel therapeutic targets and biomarkers of early atherosclerosis. The findings provide insights to deepen the understanding of molecular mechanisms that underlie early atherosclerosis in baboons, potentiating translation to humans.

## Limitations and future studies

Although our findings provide the first insights regarding miRNA and predicted miRNA targets associated with early atherosclerotic lesions, we reveal limitations that may hinder generalization. Because it was not feasible to perform RNA Seq on the FPEE samples to generate gene expression data (due to mRNA degradation), our study relied on computational prediction of miRNA targets with highly predicted confidence or experimental validation using public databases. Although we analyzed samples that are representative of each lesion type exhibited in the baboon cohort, future studies are needed to validate these findings in a larger cohort.

Our future studies will assess proteins from the CIA samples using mass spectrometry to identify expressed miRNA targets and perform molecular mechanistic studies to deepen our understanding of miRNA mechanistic roles in primate early atherosclerotic lesions.

## Supplementary Information

Supplementary Information.

## Data Availability

For the current study, datasets generated during and/or analyzed are available in Sequence Read Achieve (SRA) repository (Accession Number PRJNA883251).
