# Supplementary material for: Potential miRNA biomarkers and therapeutic targets for early atherosclerotic lesions
Source: Sci Rep. 2023 Mar 1;13:3467. doi: 10.1038/s41598-023-29074-1 (PMC9977938; doi:10.1038/s41598-023-29074-1)
Supplement: Supplementary file 1 — Supplementary Information. [file 41598_2023_29074_MOESM1_ESM.docx]

**Supplemental Table 1: miRNA targets.** Underlined are genes unique to the lesion type.

| **Fatty streak** | **cellular localization** | **Fibrious plaque** | **cellular localization** |
| --- | --- | --- | --- |
| ABCB1 | Plasma Membrane | ABCB1 | Plasma Membrane |
| AKTIP | Cytoplasm | AGO2 | Cytoplasm |
| ARID4B | Nucleus | AGO3 | Cytoplasm |
| BCL2 | Cytoplasm | AKTIP | Cytoplasm |
| BCL2L11 | Cytoplasm | CCNA2 | Nucleus |
| CXCL8 | Extracellular Space | CDKN3 | Cytoplasm |
| DNMT1 | Nucleus | COL13A1 | Plasma Membrane |
| DNMT3B | Nucleus | DTD1 | Cytoplasm |
| DUSP1 | Nucleus | ENPP6 | Cytoplasm |
| E2F2 | Nucleus | FBXO33 | Other |
| E2F3 | Nucleus | HIPK3 | Nucleus |
| EGR2 | Nucleus | LTB | Cytoplasm |
| ENPP6 | Cytoplasm | MIF | Extracellular Space |
| FBXO33 | Other | MTDH | Cytoplasm |
| GATA1 | Nucleus | POLE2 | Nucleus |
| HIPK3 | Nucleus | RAD54L | Nucleus |
| IL6 | Extracellular Space | S100A12 | Cytoplasm |
| ITCH | Nucleus | TMSB15A | Cytoplasm |
| JAK1 | Cytoplasm | TP53 | Nucleus |
| JARID2 | Nucleus | TUSC2 | Nucleus |
| MEF2D | Nucleus |  |  |
| MIF | Extracellular Space |  |  |
| MMP3 | Extracellular Space |  |  |
| NR1I2 | Nucleus |  |  |
| PURA | Nucleus |  |  |
| RBL2 | Nucleus |  |  |
| TGFBR2 | Plasma Membrane |  |  |
| TLR3 | Plasma Membrane |  |  |
| TP53 | Nucleus |  |  |
| TWIST1 | Nucleus |  |  |
| VEGFA | Extracellular Space |  |  |
| ZBTB7A | Nucleus |  |  |
